# Criterion scores, construct validity and reliability of a web-based instrument to assess physiotherapists' clinical reasoning focused on behaviour change: ‘Reasoning 4 Change’

**DOI:** 10.3934/publichealth.2018.3.235

**Published:** 2018-07-06

**Authors:** Maria Elvén, Jacek Hochwälder, Elizabeth Dean, Olle Hällman, Anne Söderlund

**Affiliations:** 1 Division of Physiotherapy, School of Health, Care and Social Welfare, Mälardalen University, Västerås, Sweden; 2 Division of Psychology, School of Health, Care and Social Welfare, Mälardalen University, Eskilstuna, Sweden; 3 Department of Physical Therapy, Faculty of Medicine, University of British Columbia, Vancouver, BC, Canada; 4 Department of Information Technology, Uppsala University, Uppsala, Sweden

**Keywords:** assessment, behaviour change, clinical reasoning, education, physiotherapy, psychometrics, reliability, validity, web application

## Abstract

*Background and aim:* ‘Reasoning 4 Change’ (R4C) is a newly developed instrument, including four domains (D1–D4), to assess clinical practitioners' and students' clinical reasoning with a focus on clients' behaviour change in a physiotherapy context. To establish its use in education and research, its psychometric properties needed to be evaluated. The aim of the study was to generate criterion scores and evaluate the reliability and construct validity of a web-based version of the R4C instrument. *Methods:* Fourteen physiotherapy experts and 39 final-year physiotherapy students completed the R4C instrument and the Pain Attitudes and Beliefs Scale for Physiotherapists (PABS-PT). Twelve experts and 17 students completed the R4C instrument on a second occasion. The R4C instrument was evaluated with regard to: internal consistency (five subscales of D1); test-retest reliability (D1–D4); inter-rater reliability (D2–D4); and construct validity in terms of convergent validity (D1.4, D2, D4). Criterion scores were generated based on the experts' responses to identify the scores of qualified practitioners' clinical reasoning abilities. *Results:* For the expert and student samples, the analyses demonstrated satisfactory internal consistency (α range: 0.67–0.91), satisfactory test-retest reliability (ICC range: 0.46–0.94) except for D3 for the experts and D4 for the students. The inter-rater reliability demonstrated excellent agreement within the expert group (ICC range: 0.94–1.0). The correlations between the R4C instrument and PABS-PT (*r* range: 0.06–0.76) supported acceptable construct validity. *Conclusions:* The web-based R4C instrument shows satisfactory psychometric properties and could be useful in education and research. The use of the instrument may contribute to a deeper understanding of physiotherapists' and students' clinical reasoning, valuable for curriculum development and improvements of competencies in clinical reasoning related to clients' behavioural change.

## 1. Introduction

With the recognition of the impact of lifestyle behaviours on individual and population health [1] and the growing evidence of behavioural considerations in physiotherapy [2],[3], there is a need to advance the clinical reasoning of physiotherapists [4] i.e., their thoughts and decisions in client management [5]. Enabling such advancements requires investigation and evaluation in physiotherapy education and practice, which in turn requires reliable and valid assessment of clinical reasoning ability. Also, assessment instruments need to capture clinical reasoning abilities that respond to the changing global health panorama and their associated priorities in professional competency through health promotion and support of health-related behaviour changes [6].

Clinical reasoning is a central competence for physiotherapists and a cornerstone in the entry-level education [7]. The importance of clinical reasoning has contributed to the development of a variety of assessment tools. Many tools used in physiotherapy education lack standardisation [7]. Furthermore, these tools may target particular areas of practice [8] or measure features of clinical competence or performance [9]–[12], but may not specifically address clinical reasoning ability across physiotherapy contexts. The newly developed clinical reasoning instrument, ‘Reasoning 4 Change’ (R4C) [13] was developed to meet these needs and shortcomings. The R4C instrument includes four domains and targets the features of a biopsychosocial clinical reasoning process with a focus on clients' activity-related behaviour and behaviour change. However, to establish its use in education and research, further evaluation is warranted.

The R4C instrument has been built on a solid theoretical base that has been described previously in the clinical reasoning model focused on clients' behaviour change with reference to physiotherapists (CRBC-PT) [14]. The nature of the clinical reasoning is characterised by a cognitive, reflective, contextual and collaborative reflexive process with multiple interrelated levels incorporating the client's behaviours and goals, behaviour analysis and behaviour change strategies. The original development of the R4C instrument followed a detailed stepwise process [13] including consideration of current guidelines for clinical reasoning assessment design. For example, features of the design included multiple cases based on the role of case specificity [15], the addition of new client information throughout the reasoning process [16] and multiple acceptable reasoning paths [17]. The process resulted in a paper-based version of the R4C instrument. Web-based formats are effective and feasible [18],[19] and have been proposed to better reflect simulations of real-life clinical case scenarios than paper-based formats can provide [20]. A shift from hard copy to a web-based application was therefore deemed essential to develop a sound easily applicable instrument.

Script concordance testing is an established method to assess clinical reasoning [21] and has informed the R4C instrument. The method is based on the principle that clinical reasoning ability is founded in knowledge structures (scripts) that develop and reorganise in response to extended experiences [22]. Thus, experts' scores on clinical reasoning instruments may provide an indication of clinical reasoning quality [19]. Test scores of experienced physiotherapists who specialise in a behavioural medicine approach may represent superior clinical reasoning ability, thus could serve as examples for others without such specialisation. Experienced professionals commonly differ in their decision-making processes of complex problems which may result in several acceptable solutions [23]. Thus, scoring in clinical reasoning instruments should consider the normal variability of responses from experts when scores are provided to professionals with less knowledge and experience [24].

Feasibility and comprehensibility of the R4C instrument have been supported by physiotherapy students, and its content validity has been accepted by experts in physiotherapy and behavioural medicine [13]. However, information regarding R4C instrument criterion scores, i.e., expert scores that correspond to qualified practitioners' clinical reasoning abilities, reliability and construct validity is lacking. Part of the construct validation process involves assessment of convergent validity, or similarity of constructs [25]. Associations between relevant outcomes of the R4C instrument and the Pain and Beliefs Scale for physiotherapists (PABS-PT), a measure that captures biopsychosocial and biomedical treatment approaches [26],[27] could support construct validation. The *a priori* hypotheses for construct validity were: there are strong associations between the scores of D1.4, D2 and D4 and the biopsychosocial subscale of the PABS-PT; and there are weak associations between the scores of D1.4, D2 and D4 and the biomedical subscale of the PABS-PT.

The general aim of the study was to generate criterion scores and evaluate the reliability and construct validity of a web-based version of the R4C instrument. Specifically, the aims were: (1) to generate criterion scores based on the responses to the items of the R4C instrument by a cohort of physiotherapy experts; (2) to assess the reliability of the R4C instrument for physiotherapy experts and students in terms of internal consistency, test-retest reliability and inter-rater reliability; and (3) to assess construct validity for physiotherapy experts and students in terms of convergent validity.

## 2. Materials and methods

### 2.1. Design

This study had a descriptive design for collecting and describing demographic data and generating criterion scores, and a correlational design for evaluating reliability and validity.

### 2.2. Ethics

This study was reviewed by the Regional Ethical Review Board, Uppsala, Sweden, and met the ethical requirements consistent with Swedish law (SFS 2003:460) and the Helsinki declaration [28] related to human research (Dnr 2013/020). Written consent was obtained from the participants.

### 2.3. Participants

#### 2.3.1. Experts

Twenty physiotherapists with expertise in behavioural medicine fulfilled the inclusion criteria and were invited to participate in the study. Fourteen of these participated on the first occasion of the administration of the R4C instrument and twelve participated in the test-retest reliability of the instrument. Formation of the expert sample followed the recommendation of including more than ten experts for score generation in new clinical reasoning instruments [29]. More experts than recommended were included to adjust for possible dropouts. Furthermore, experts should be credible, which implies consistency with the goal of the study [19] and fulfillment of relevant knowledge and experience attributes [30]. In the current study, individuals were defined as experts if they fulfilled the following criteria: (a) registered physiotherapist in Sweden; (b) with a PhD or currently enrolled as a PhD student in physiotherapy; (c) conduct research within physiotherapy with a behavioural medicine approach; (d) have academic qualifications that include at least five weeks of full-time studies in behavioural medicine at the postgraduate level or experience in teaching in related content; and (e) experience in teaching clinical reasoning. The experts were identified through the research team's network along with a snowball-sampling strategy [31]. The reasons for non-participation were lack of time (*n* = 4), self-identified as having insufficient knowledge (*n* = 1), on sick leave (*n* = 1), and no reason (*n* = 2 at the second occasion). The experts were faculty members at five universities in Sweden. Sample characteristics are presented in Table 1.

#### 2.3.2. Physiotherapy students

Seventy-one physiotherapy students in their final semester at two undergraduate entry-level physiotherapy programmes in Sweden were invited to participate in the study. The universities that housed the physiotherapy programmes were located in two medium-sized cities. Thirty-nine students participated, resulting in a response rate of 54% (18 from University A and 21 from University B). The students stated lack of time and heavy study load as the major reasons for not participating. Seventy-seven per cent of the participants were women and 23% were men, with an average age of 24 y (*SD* = 3.4; min 22 y, max 39 y). Seventeen students from University A participated in the test-retest reliability of the instrument.

There were no significant differences between the physiotherapy students from University A and University B regarding gender, age, work experiences in the area of health and welfare or other areas, ongoing clinical placement in the physiotherapy programme, studies other than physiotherapy, study break, or experience of teaching peers during their education. Consequently, the 39 physiotherapy students were treated as one sample. Sample characteristics are presented in Table 2.

**Table 1.** Demographic characteristics of the physiotherapy experts (*n* = 14).

Characteristics	*n*	(%)	*M*	*SD*
Gender				
Female	12	(86)		
Male	2	(14)		
Age (y)			50	9.6
30–39	3	(21)		
40–49	3	(21)		
50–59	5	(36)		
60–69	3	(21)		
Academic qualification				
PhD student	5	(36)		
PhD	7	(50)		
Associate professor	1	(7)		
Professor	1	(7)		
Current clinical practice	4	(29)		
Current teaching practice	13	(93)		
Experience in teaching clinical reasoning (years)			11	9.5
2–3	3	(21)		
4–5	2	(14)		
6–10	3	(21)		
11–20	4	(29)		
21–35	2	(14)		
Experience in research within physiotherapy with a behavioural medicine approach (years)			7.6	4.2
3–5	6	(43)		
6–10	4	(29)		
11–15	3	(21)		
>15	1	(7)		
Studies in behavioural medicine at postgraduate level or experience of teaching in such courses (credits^a^)			16	13.3
7.5	5	(36)		
>7.5–15	8	(57)		
>15	1	(7)		

^a^1.5 credits correspond to one week of full-time studies.

**Table 2.** Demographic characteristics of the physiotherapy students (*n* = 39).

Characteristics	*n*	(%)	*M*	*SD*
Gender				
Female	30	(77)		
Male	9	(23)		
Age (y)			24	3.4
22–24	28	(72)		
25–29	9	(23)		
30–34	1	(2.5)		
35–39	1	(2.5)		
Work experience in the area of health and welfare				
Yes	11	(28)		
No	28	(72)		
Work experience in other areas				
Yes	35	(90)		
No	4	(10)		
On-going clinical placement in the physiotherapy program				
Yes	0	(0)		
No	39	(100)		
Studies other than physiotherapy (credits ^a^)			1.2	1.6
No	20	(51)		
> 0–7.5	8	(21)		
> 7.5–30	6	(15)		
> 30–120	4	(10)		
> 120	1	(3)		
Experience of teaching peers				
Yes	4	(10)		
No	35	(90)		

^a^ 1.5 credits correspond to one week of full-time studies.

### 2.4. Measures

#### 2.4.1. The ‘Reasoning 4 Change’ (R4C) Instrument

Physiotherapists' clinical reasoning focused on clients' activity-related behaviour and behaviour change was assessed with the R4C instrument. A detailed description of the theoretical foundation, development process, feasibility and content validation of the R4C instrument has been published previously [13]. The R4C instrument is multidimensional, consistent with the various dimensions of the CRBC-PT model [14]. The instrument consists of four domains: Physiotherapist domain (D1) including five subscales; Knowledge (D1.1), Cognition (D1.2), Metacognition (D.1.3), Psychological factors (D1.4), and Contextual factors (D1.5), Input from client domain (D2), Functional behavioural analysis domain (D3), and Strategies for behaviour change domain (D4). The subscales of D1 are based on self-assessments and include 49 items in total. The items of the subscales are made up of statements that the examinee judges on either 6-point or 11-point Likert scales. D2, D3, and D4 consist of written case scenarios, eight in total, which are gradually extended with new information. D2 includes 12 items, D3 eight items, and D4 12 items. Each of these items could consist of a single item with up to six sub-items. These items assess the examinee's ability to identify, prioritise, analyse and interpret key features that pertain to components in the management of the case. Key features comprise critical stimuli that can be used to solve a clinical problem [32]. These stimuli may be descriptions of activity and participation problems, unhealthy lifestyle-related behaviours, associations among physical, psychological, and contextual factors, hypotheses explaining factors that potentially cause, control or maintain a target behaviour, or data from assessments and analyses guiding decisions and actions aimed to support behaviour change. The response scales of D2, D3, and D4 include Likert scales, write-in formats, and lists of options. An overview of the domains of the R4C instrument and characteristics of cases, items, and response scales has been documented previously [13]. Examples of the four domain items are shown in Supplement A.

##### (1) The web-based application

The paper-based R4C instrument [13] was transferred to a web-based application by a team that included the instrument's investigators, a software developer, and a senior researcher with expertise in computer science and specialisation in interactive design. A specification for the design and system requirements of the application was developed based on the theories and evidence underpinning the R4C instrument [13],[14] and guidelines regarding layout and function [33],[34] as shown in Supplement B. Seven individuals tested the beta (preliminary) version of the web application to detect software bugs and receive feedback about layout and function. A few software bugs were identified and feedback was given regarding a few ambiguous instructions. These were corrected and clarifications were made before the web-based version was released. This version of the R4C instrument was used in the current study. Features of the web-based R4C application included instructions for responding and definitions of key concepts; the items of domains one, two, three, and four; an administration tool; and a tool for compilation of scoring.

##### (2) The scoring key

In D1, the score for each item corresponded to the examinee's chosen response option on the Likert scales. The item scores were totalled for each subscale. The scoring key of D2–D4 was based on the scoring method of the Script concordance test, in which scores are derived from responses given by an expert panel [19]. An aggregate method [35] was used to reflect the fact that professionals' decision paths vary [23]. In other words, the responses of any expert were taken into account, and responses with poor agreement among experts were not discarded. In the current study, the sample of physiotherapy experts (*n* = 14) comprised the expert panel. Scores for items with response options (lists of options and Likert scales) were computed from the numbers of experts that chose a specific response option for an item, divided by the modal value for that item [19],[35]. For example, for a given item, if ten experts chose response option ‘+2’, three chose ‘−1’ and one chose ‘0’, the result was a modal value of 10, thus the score for the ‘+2’ was one point (10/10), the score for the ‘−1’ was 0.3 points (3/10), and the score for ‘0’ was 0.1 points (1/10). Response options that were not chosen by any expert received zero points. D2 and D4 include two items each with write-in answers. The scores of these items were initially based on qualitative judgements and categorisation of the experts' responses, independently conducted by two of the investigators (M.E. and A.S.). Consensus was achieved in dialogue. Subsequently, based on the frequencies of the experts' responses within the categories, the scores were generated according to the aggregate scoring method. For two items of D4, in which the importance of biopsychosocial factors should be assigned in percentage, the scores were derived from the experts' means and standard deviations. The item scores were totalled for D2, D3, and D4 separately.

Min–max scores for the items and domains varied and were dependent upon item characteristics and number of items per domain. The instrument's composition resulted in eight total scores: five total scores for the subscales of D1, and three total scores for D2, D3, and D4. Higher scores indicate a higher degree of clinical reasoning ability, which supports a focus on clients' activity-related behaviour and behaviour change.

#### 2.4.2. The Pain Attitudes and Beliefs Scale for Physiotherapists (PABS-PT)

Biomedical and biopsychosocial treatment orientations were assessed with the PABS-PT [26],[27]. The PABS-PT is a self-administered questionnaire designed to assess two treatment orientations towards management of patients with non-specific persistent low back pain. The original PABS-PT consisted of 20 items divided into two factors (biomedical and biopsychosocial orientations) [27]. Further testing of factor structure confirmed a two-factor solution which resulted in an amended version of 19 items (ten biomedical and nine behavioural items) [26]. In the present study, a Swedish translation of the 19-item questionnaire was used [36]. Scores on the Biomedical subscale range from ten to 60 and the Biopsychosocial subscale scores range from nine to 54, with a higher score indicating stronger treatment orientation. No consensual cut-offs for high or low scores have been reported. The PABS-PT has demonstrated satisfactory construct validity, test-retest reliability and responsiveness when tested in different contexts [37]. Internal consistency assessed with Cronbach's alpha ranged from 0.73 to 0.84 for the biomedical factor and 0.54 to 0.68 for the biopsychosocial factor [26],[27],[37],[38].

### 2.5. Procedures

#### 2.5.1. Testing procedures

Qualified experts were invited to participate in the current study. Upon acceptance of invitation, the experts received the PABS-PT and a web-link to the R4C instrument, along with a letter explaining the aim and procedure of the study and individual log-in information through e-mail. The experts completed the R4C instrument on a computer or a tablet and the PABS-PT in hard copy. The experts were asked to complete the assessment within two weeks. To investigate test-retest, a new web-link to the R4C instrument was e-mailed to the experts two weeks after their first response. The mean time interval for responses in the test-retest investigation was 24 days (range = 15 to 40 days). The experts' (*n* = 14) responses on the first test occasion provided the basis for the scoring key of D2–D4 [19] and the generation of criterion scores for D1–D4 [25].

The physiotherapy students' participation in the study was approved by the directors of their programmes. The data collection took place in a designated room at the students' university that was equipped with computers and lasted for two hours. Verbal and written information about the aim and procedure of the study was provided by the primary investigator, who was present during the data collection. The session began with the students providing demographical data and the completion of the PABS-PT in hard copy. Then the students were given a web-link to the R4C instrument and individual log-in information. Collaboration among the students was not permitted. Three weeks after the first occasion, due to the students' schedule, the students from University A completed the R4C instrument a second time. Five students completed the R4C instrument in a lecture hall and 12 on private computers due to unforeseen schedule changes. The mean time interval for responses in the test-retest investigation was 20 days (range: 15–37 days).

### 2.6. Statistical analyses

The analyses were carried out with the IBM SPSS Statistics for Macintosh, Version 22.0 (Armonk, NY: IBM Corp). For demographic data, frequencies, means, standard deviations and ranges were used as descriptive measures. Differences in demographic variables between the physiotherapy students from Universities A and B were analysed with Pearson's chi-square test or Fisher's exact test for categorical variables and independent t-tests for continuous variables [39]. Distributional properties in the forms of means, standard deviations, medians, quartiles, ranges, skewness and kurtosis were calculated for each domain and subscale of the R4C instrument and PABS-PT. Z-scores of skewness and kurtosis greater than 1.96 indicated a significant (*p* < 0.05) asymmetric distribution (skewness) or an either more or less clustered distribution score around its central point (kurtosis) [39]. Criterion scores in the forms of means, standard deviations, medians, quartiles and ranges were calculated for each domain and subscale.

#### 2.6.1. Reliability

Reliability was assessed in terms of internal consistency, test-retest reliability and inter-rater reliability. Internal consistency was calculated with Cronbach's alpha [40] for each subscale of D1. Cronbach's alpha coefficients ≥ 0.70 [41] were considered to be satisfactory and coefficients ≥ 0.90 [42] were considered as an indicator of redundancy.

Test-retest reliability [25] was analysed for the total scores of the five subscales of D1, and for D2, D3, and D4. Inter-rater reliability [43] was analysed between the 14 experts' independent responses and between all possible combinations of expert pairs for D2, D3, and D4. Test-retest and inter-rater reliability were calculated using the Intra Class Correlation coefficient (ICC) with two-way mixed model, absolute agreement, and average measures [44]. Agreement was interpreted as poor for ICC values less than .40, fair for values between .40 and 0.59, good for values between 0.60 and 0.74, and excellent for values above 0.75 [45]. Homogeneity of variance was checked by computing the standard deviation ratio of the second and first test scores for the subscales and domains, respectively. A ratio around 1.0 was interpreted as acceptable [44].

#### 2.6.2. Validity

Convergent validity, a form of construct validity that shows the extent to which various measures of theoretically similar constructs correlate with one another [25],[46] was calculated using the Pearson product-moment correlation coefficient. The analyses included correlations between the outcomes of D1.4, D2, and D4 of the R4C instrument and the biopsychosocial subscale of the PABS-PT for experts and students separately and correlations between the outcomes of D1.4, D2, and D4 of the R4C instrument and the biomedical subscale of the PABS-PT for experts and students separately. Altogether, 12 analyses of correlation were conducted. There is no established cut-off value defining convergent validity, as the associations between constructs should be judged according to predefined hypotheses [25]. According to DeVon et al. [46], a correlation coefficient of *r* ≥ 0.45 is an accepted standard of a high correlation. If multiple hypotheses are stated in advance and at least 75% of the results are in accordance with these hypotheses, convergent validity should be interpreted as satisfactory [47].

## 3. Results

The score distributions for the R4C instrument and PABS-PT for the physiotherapy experts (*n* = 14) and students (*n* = 39) are presented in Tables 3 and 4, respectively. For the experts, no domains or subscales significantly differed from normality according to skewness and kurtosis, except for one (D2). D2 was significantly and negatively skewed (skewness = −1.41; z-score of skewness = 2.37; *p < 0.05*). For the students, no domains or subscales significantly differed from normality.

The criterion scores for the domains and subscales of the R4C instrument are presented in Table 3.

**Table 3.** Score distributions and criterion scores for the R4C instrument, and score distributions for the PABS-PT for the experts (*n* = 14). Theoretical min-max total scores, Means (*M*), standard deviations (*SD*), medians (*Mdn*), first and third quartiles (*Q*_1_ and *Q*_3_), and observed min-max scores of the domains and subscales.

R4C instrument: Domain and subscale	Theoretical min-max total scores	*M*	*SD*	*Mdn*	*Q*_1_	*Q*_3_	Observed min–max scores
D1.1 Physiotherapist; Knowledge	8–48	39.0	5.3	39.0	34.8	44.3	32–48
D1.2 Physiotherapist; Cognition	7–46	36.9	5.5	36.0	31.8	42.3	30–46
D1.3 Physiotherapist; Metacognition	8–48	43.0	3.8	43.0	40.8	46.3	36–48
D1.4 Physiotherapist; Psychological factors	0–200	181.3	12.3	181.5	172	194.5	158–198
D1.5 Physiotherapist; Contextual factors	5–30	17.9	6.4	16.5	13.5	23.0	8–29
D2 Input fromclient	0.6–66.1	52.2	5.1	53.2	51.2	54.6	40.3–58.2
D3 Functional behavioural analysis	3.8–34.3	28.2	1.8	28.2	27.2	29.2	24.7–31.7
D4 Strategies for behaviour change	0–36.4	23.7	2.4	23.5	22.6	25.9	18.4–27.6
PABS-PT Biopsychosocial	9–54	42.4	3.3	43.0	39.0	45.3	37–47
PABS-PT Biomedical	10–60	24.9	5.0	25.0	21.0	28.3	15–35

**Table 4.** Score distributions for the R4C instrument and the PABS-PT for the physiotherapy students (*n* = 39). Theoretical min-max total scores, means (*M*), standard deviations (*SD*), medians (*Mdn*), first and third quartiles (*Q*_1_ and *Q*_3_), and observed min-max scores of the domains and subscales.

R4C instrument: Domain and subscale	Theoretical min-max total scores	*M*	*SD*	*Mdn*	*Q*_1_	*Q*_3_	Observed min-max scores
D1.1 Physiotherapist; Knowledge	8–48	36.0	4.0	35.0	34.0	39.0	27–44
D1.2 Physiotherapist; Cognition	7–46	34.6	5.1	35.0	32.0	38.0	23–44
D1.3 Physiotherapist; Metacognition	8–48	38.7	4.4	39.0	34.0	43.0	29–45
D1.4 Physiotherapist; Psychological factors	0–200	149.4	18.0	148.0	138.0	161.0	116–185
D1.5 Physiotherapist; Contextual factors	5–30	17.5	4.5	17.0	14.0	21.0	11–28
D2 Input from client	0.6–66.1	37.7	5.8	37.2	32.6	41.3	27.1–50.2
D3 Functional behavioural analysis	3.8–34.3	24.4	2.8	24.7	22.3	26.6	16.9–30.0
D4 Strategies for behaviour change	0–36.4	19.4	3.2	18.9	17.2	21.9	13.0–25.7
PABS-PT Biopsychosocial	9–54	39.3	3.4	40.0	37.0	42.0	33–47
PABS-PT Biomedical	10–60	33.8	7.4	33.0	29.0	38.0	20–52

### 3.1. Reliability

#### 3.1.1. Experts

The internal consistency was satisfactory for all subscales of D1 for the experts (*n* = 14). The Cronbach's α coefficients were D1.1 = 0.86; D1.2 = 0.88; D1.3 = 0.67; D1.4 = 0.88; and D1.5 = 0.84.

The standard deviation ratios ranged between 0.8 and 1.5, for the experts, judged as acceptable homogeneity of variance. Test-retest analyses of total scores of the subscales of D1 showed significant and excellent agreement regarding the ICC coefficients (ICC range: 0.75–0.89). The ICCs for D2, D3, and D4 showed poor or fair agreement (ICC range: 0.31–0.45). After exclusion of the most prominent outlier (the expert with the largest score difference between tests 1 and 2), the agreements were fair or good (ICC range: 0.46–0.61). Detailed results of test-retest reliability for the experts are presented in Table 5.

The inter-rater reliability for D2, D3 and D4, calculated with ICCs demonstrated excellent agreement within the expert group (*n* = 14): D2 (12 items) ICC = 1.0 (*p* < 0.001); D3 (8 items) ICC = 0.99 (*p* < 0.001); and D4 (12 items) ICC = 0.94 (*p* < 0.001). Analyses of all possible paired combinations of the experts showed ICCs ranging between 0.89 and 1.0 for D2, 0.60 to 0.98 for D3, and 0.15 to 0.94 for D4.

#### 3.1.2. Physiotherapy students

The internal consistency was satisfactory for all subscales of D1 for the physiotherapy students (*n* = 39). The Cronbach's α coefficients were; D1.1 = 0.74; D1.2 = 0.84; D1.3 = 0.80; D1.4 = 0.91; and D1.5 = 0.75.

The standard deviation ratios ranged between 0.8 and 1.3 for the physiotherapy students, judged as acceptable homogeneity of variance. Test-retest analyses of total scores of the subscales of D1 showed significant and excellent agreement regarding the ICCs (ICC range: 0.81–0.92). The ICCs for D2, D3, and D4 showed fair or good agreement (ICC range: 0.45–0.72). After exclusion of the most prominent outlier (the physiotherapy student with the largest score difference between test 1 and 2) in D4, the ICC was 0.55. Detailed results of test-retest reliability for the students are presented in Table 5.

**Table 5.** Test-retest reliability for the physiotherapy experts (*n* = 12) and the physiotherapy students (*n* = 17).

R4C instrument: Domain and subscale	Physiotherapy experts				Physiotherapy students			
	*M (SD)* Test 1	*M (SD)* Test 2	ICC ^a^	ICC ^a,b^	*M (SD)* Test 1	*M (SD)* Test 2	ICC ^a^	ICC ^a,b^
D1.1 Physiotherapist; Knowledge	38.5 (5.0)	39.5 (4.8)	0.89***		37.4 (4.5)	38.8 (4.2)	0.87***	
D1.2 Physiotherapist; Cognition	37.3 (5.5)	38.6 (4.9)	0.94***		36.4 (5.1)	35.7 (4.6)	0.87***	
D1.3 Physiotherapist; Metacognition	43.3 (3.4)	43.5 (3.8)	0.75*		39.1 (5.2)	39.4 (5.0)	0.92***	
D1.4 Physiotherapist; Psychological factors	182.2. (10.6)	177.6 (13.7)	0.89***		153.4 (17.8)	154.6 (18.7)	0.85***	
D1.5 Physiotherapist; Contextual factors	17.6 (5.7)	19.7 (4.5)	0.77**		15.1 (3.9)	16.4 (3.2)	0.81***	
D2 Input from client	53.0 (4.1)	44.5 (6.3)	0.31	0.46*	40.6 (5.8)	39.2 (5.0)	0.72**	
D3 Functional behavioural analysis	27.9 (1.6)	28.3 (2.3)	0.31	0.56	26.0 (2.2)	25.4 (1.9)	0.60*	
D4 Strategies for behaviour change	24.1 (2.1)	21.3 (2.8)	0.45	0.61*	21.0 (3.2)	21.5 (2.8)	0.45	0.55

^a^ Two-way mixed model, absolute agreement and average measure.

^b^ One outlier (the expert or student with the largest score difference between test one and two was excluded in the analysis).

* p < 0.05; ** p < 0.01; ***p < 0.001.

### 3.2. Validity

#### 3.2.1. Experts

The analyses of correlation between the scores of the R4C instrument and the PABS-PT confirmed six out of six (100%) *a priori* stated hypotheses for the expert group. The confirmed hypotheses were: strong associations between the scores of D1.4, D2, and D4 and the biopsychosocial subscale of the PABS-PT; and weak associations between the scores of D1.4, D2, and D4 and the biomedical subscale of the PABS-PT. Detailed results are presented in Table 6.

#### 3.2.2. Physiotherapy students

The analyses of correlation between the scores of the R4C instrument and the PABS-PT confirmed three out of six (50%) *a priori* stated hypotheses for the student group. The confirmed hypotheses were: weak associations between the scores of D1.4, D2, and D4 and the biomedical subscale of the PABS-PT. The non-confirmed hypotheses were: strong associations between the scores of D1.4, D2, and D4 and the biopsychosocial subscale of the PABS-PT. Detailed results are presented in Table 6.

Altogether, nine out of 12 (75%) specific hypotheses for the physiotherapy experts and students were confirmed, which was judged as acceptable construct validity.

**Table 6.** Convergent validity. Pearson's product-moment correlation coefficients (*r*) for the domains and subscales of R4C and the two subscales of PABS-PT for the physiotherapy experts (*n* = 14) and students (*n* = 39).

R4C instrument: Domain and subscale	Physiotherapy experts		Physiotherapy students	
	PABS-PT-BPS	PABS-PT-Biomed	PABS-PT-BPS	PABS-PT-Biomed
	*r*	*r*	*r*	*r*
D1.4 Psychological factors	0.59*	0.09	−0.06	−0.15
D2 Input from client	0.76***	−0.10	0.20	−0.38**
D4 Strategies for behaviour change	0.73***	−0.17	−0.07	−0.21

* p < 0.05 (one-tailed); ** p < 0.01 (one-tailed); ***p < 0.001 (one-tailed).

## 4. Discussion

The psychometric evaluation of the web-based R4C instrument demonstrated satisfactory reliability in terms of internal consistency, test-retest reliability, and inter-rater reliability and acceptable construct validity in terms of convergent validity. The described score distributions of the physiotherapy students and the generated criterion scores, based on the experts' responses, are useful for interpretations of future assessments of students' and practitioners' clinical reasoning assessed with the R4C instrument.

The score distributions of the physiotherapy experts and the students revealed that there was an overrepresentation of responses in the upper half of the scales. Similar score distributions have been demonstrated in Script concordance tests used to assess clinical reasoning of experts and students in medical and nursing education [48], and has allowed detection of changes in competence [49],[50]. In the current study, the score distribution of D2 was negatively skewed for the experts, but not for the students. Thus, one ceiling effect emerged. This result indicates that the instrument has potential to capture improvement and deterioration in physiotherapists' and students' clinical reasoning ability.

### 4.1. Criterion scores

The aggregate scoring method is the most established method used for evaluation of clinical reasoning abilities. However, other scoring methods exist (e.g. the consensus method), and the most optimal method in terms of reliability and validity is still debated [21],[51]. The aggregate scoring method has been reported to be less reliable (lower Cronbach's α coefficient) but more valid than the consensus method as it allows finer discrimination between experts and students, implying greater construct validity [35]. On the other hand, Lineberry, Kreiter, and Bordage [52] questioned the Script concordance test's validity when the aggregate scoring method was practiced. They highlighted the risk of introducing logical inconsistences into the scoring key when all responses were judged as ‘correct’. Despite identified shortcomings of the aggregate scoring method, current guidelines for the Script concordance test construction [19],[24],[53] and literature reviews [21],[54] still propose this method as the preferred choice. These results informed the choice of scoring method for the R4C instrument. Gagnon et al. [29] demonstrated that an expert panel of any number over ten members was associated with acceptable reliability for assessment of clinical reasoning based on the aggregate scoring method. Thus, the sample size of 14 experts was considered appropriate for the construction of the scoring key of the R4C instrument.

Epistemological viewpoints of clinical reasoning also support a range of acceptable paths in client encounters. Clients are managed within a rich context and each professional brings his or her experience to the situation and make his or her own interpretation [17]. Empirical evidence of factors that influence physiotherapists' clinical reasoning strengthens the belief that clinical decisions depend upon the context, individual and situation, resulting in variability of decision paths [55]. This is demonstrated for both experienced and novice physiotherapists [56]. Accordingly, in the current study, responses of the experts were considered valid and all response options selected by one or more experts generated a partial credit. Even though experts differ in the decisions made in a reasoning process, their answers usually converge towards a similar outcome [35]. According to the ICCs for inter-rater reliability this was the case in current study. The experts' responses displayed the recommended balance of unanimity and moderate response distribution [53], which strengthened the reliability of the criterion scores.

Physiotherapists need to be equipped with adequate competencies in order to contribute to a healthier lifestyle in the population and reduce the prevalence of non-communicable diseases [4],[6]. Such prioritised competencies embrace the adoption of a biopsychosocial model of health and functioning as a frame of reference in client care [57] and the integration of evidence-based behaviour change strategies into physiotherapy practice [2],[3],[58]. Even though the biopsychosocial perspective has vastly expanded in physiotherapy, a biomedical perspective still exists and sometimes even dominates clinical reasoning [59],[60]. Furthermore, strategies to support behaviour change seldom influence the reasoning process [55]. Given this picture, entry-level and further continuing professional education are indicated to better prepare physiotherapists in dealing with psychosocial complexities of contemporary health conditions [61] and mastery of effective behaviour change skills [4],[62]. Physiotherapists with recognised behavioural medicine expertise may best support such competency development. The characteristics of their clinical reasoning process may serve as benchmarks for effective reasoning. Accordingly, the established criterion scores for the R4C instrument are valuable in order to make sense of physiotherapists' and students' scores and to improve accuracy in conclusions about their clinical reasoning abilities to support behaviour change.

### 4.2. Reliability

Dimensionality, reliability and validity are interrelated measurement properties, thus need to be carefully considered in instrument development and evaluation [25],[63]. The theoretical dimensionality of physiotherapists' clinical reasoning focused on clients' activity-related behaviour and behaviour change delineates multiple dimensions, including several constructs [13],[14]. When a scale is multidimensional and the dimensions are made up of more than one construct, internal consistency is not relevant. In these cases, some degree of heterogeneity among items is expected [42],[64]. Accordingly, it was not appropriate to calculate α coefficients for D2, D3, and D4 in the current instrument evaluation. On the other hand, the items of the five subscales of D1 represent five distinct constructs included in the Physiotherapist dimension, therefore assessments of internal consistency were applicable. As the R4C instrument represents multiple dimensions, use of a total score was not considered meaningful [65]. Instead, distinct scores of the subscales and domains of R4C instrument were found to be appropriate.

Internal consistency for the subscales in D1 was satisfactory, indicating that the items of the subscales fit together conceptually [46]. Streiner [42] contends that α values ≥ 0.90 can represent redundancies suggesting the scale could be shortened. In the current study, the D1.4 had the highest α values, experts α = 0.88 and physiotherapy students α = 0.91, which indicated a minimal risk of overlapping items; thus, item elimination was not required.

Test-retest reliability for D2, D3, and D4 were interpreted as poor or fair for the experts, and fair and good for the physiotherapy students. These findings could be explained in several ways. First, a low ICC could be explained by a small variance in test scores between individuals [25]. Based on the restricted inclusion criteria, the expert group could be considered as a homogenous sample that resulted in similar response patterns and small variance. Second, due to the limited sample size, consistency in the participants' scores from test to retest may be sensitive to large differences. Therefore, for the domains with non-significant ICCs, the most extreme outlier of the participants was identified and excluded from the analysis. For the experts, this operation resulted in significant fair and good correlations for D2 and D4. For D3, there was a tendency towards a fair correlation; however, it was not significant. For the physiotherapy students, the ICC for D4 increased and indicated a fair correlation, but it was not significant. Third, systematic bias [41] may have occurred in D2, D3, and D4. These domains include multiple item constructions, ranging from simple to complex. For example, the three-pronged items in D3 and D4, based on the model of the Script concordance test, assess higher-order cognitive skills [19] and might be perceived as complex and demanding. It is likely that the examinees became more confident in their understanding of this item construction as they proceeded throughout the test, which could have affected their responses. This learning effect may have persisted in the second test occasion and could have resulted in systematic disagreement in responses between the test occasions. In future use of the R4C instrument, it is important to ensure that the examinees are familiar with item construction. If not, specific strategies such as preparation items could be implemented to ensure sufficient skills. In sum, the results of the current study demonstrated excellent test-retest reliability for D1 and acceptable test-retest reliability for D2-D4. Understanding of item construction is considered important to strengthen reliability.

Inter-rater reliability [43] was analysed to reveal the extent of discrepancies in the experts' responses, which was important for interpretation of the scoring key in D2–D4. The 14 experts' responses correlated to a great extent, even though individual differences were observed among the experts. The ICCs verified that the expert sample shared a common view of the reasoning processes. Hence, the results substantiated the scoring method of the R4C instrument.

### 4.3. Validity

A biopsychosocial clinical reasoning process focusing on clients' activity-related behaviour and behaviour change has similarities to a treatment approach focusing on biopsychosocial factors. Therefore, strong associations between the scores of the Biopsychosocial subscale of the PABS-PT and the R4C instrument were hypothesised. A pronounced biomedical treatment approach is not compatible with a biopsychosocial clinical reasoning process. However, biomedical factors must be considered and intertwined with psychosocial factors in order to make informed decisions in assessment and treatment. Thus, weak associations between the Biomedical subscale of the PABS-PT and the R4C instrument were hypothesised. The appearance of a biopsychosocial or biomedical approach in the clinical reasoning process is detectable primarily in D2 and D4 as these domains comprise identification and interpretation of bio-, psycho-, and social factors. Furthermore, physiotherapists' attitudes and beliefs towards a biomedical or biopsychosocial approach have been reported to be associated with certain decisions in the reasoning process [66],[67]. Accordingly, the scores of D1.4, D2 and D4 were found appropriate for the analysis of convergent validity. The reason to exclude D3 in the analysis was due to the content and construction of its items. In D3, analytical capability is the focus and the examinee's emphasis on bio-, psycho-, or social factors depends primarily on the given information and less on his or her treatment approach. Identifying a correlation coefficient as sufficient or appropriate for convergent validity evidence has been demonstrated to be difficult because no criterion standards exist [46] and other methods have been advocated for quality criteria [47]. In the current study, 75% of the predefined hypotheses were confirmed for the experts and students together, which is judged as acceptable. However, a smaller proportion of the predefined hypotheses were confirmed for the students compared to the experts. For the students, the hypotheses regarding weak associations between the scores of D1.4, D2, and D4 and the biomedical subscale of PABS-PT were confirmed but not the strong associations between the scores of D1.4, D2, and D4 and the biopsychosocial subscale of PABS-PT. A likely explanation is that a biopsychosocial approach may be more complex and challenging to apply than a biomedical approach. Thus, students may have a less established biopsychosocial treatment orientation than experienced physiotherapists, which may have contributed to the low correlations between D1.4, D2, and D4 and the biopsychosocial subscale of PABS-PT for the students. However, no conclusions about causality could be drawn from the current study. For the experts only, 100% of the predefined hypotheses were confirmed, which is a strength, since the experts served as models of qualified clinical reasoning ability. Thus, evidence for convergent validity of the R4C instrument was supported.

Cook and Beckman [68] emphasise that careful attention should be given to five sources of validity evidence in instrument development and evaluation: content, response process, internal structure/reliability, relations to other variables, and consequences. At present, four of these sources have been targeted and evaluated. Two of the sources, content validity and the comprehensibility of the response process, have previously been demonstrated to be satisfactory [13]. Because reliability is a prerequisite for validity, the findings of the current study contributed to validity evidence by evaluating internal consistency and reliability. Furthermore, the relationship between clinical reasoning focusing on behaviour change and similar constructs were investigated in the current study. The fifth source refers to predictive validity; for example, to explore the consistency between expected outcomes and achieved outcomes assessed with the instrument. Suggested next steps may be validation with contrasted groups [46] and investigations using the R4C instrument in intervention studies and in various contexts. Currently, the accomplished stepwise appraisal of evidence for validity and reliability of the R4C instrument is a strength and provides a solid foundation for further research.

### 4.4. Limitations

This study has several limitations. First, the sample sizes of experts and physiotherapy students could be considered as small [69]. The sample of students consisted of students from two of the eight physiotherapy programmes in Sweden. Thus, there is a risk that the participating students may not completely represent the student population, which needs to be considered. Furthermore, with small sample sizes, there is a risk of low variability between the individuals, resulting in lower reliability coefficients [25]. This might have been the case for both the student and expert groups. Second, the circumstances for completing the R4C instrument changed between test occasions one and two for the students. On the first occasion, the students convened in a lecture hall at the university but, on the second occasion, about two thirds of the students completed the R4C instrument on a private computer. As test scores depend on the context in which the instrument is used [25],[70], this change in procedure may have biased the test-retest results. A third limitation concerned the high means of some subscales in D1. The results reflected that the experts and students judged their knowledge, cognitive capabilities and skills and psychological influences on clinical reasoning as very good. However, there may be a risk that their responses were influenced by social desirability, which needs to be considered when interpreting the results [63]. Cautionary instructions were used about responding as honestly as possible and minimising socially desirable responses.

### 4.5. Implications

There is a need for a reliable and valid instrument to assess clinical reasoning especially in relation to clients' behaviour change in physiotherapy practice and professional education. Given the results of this study, the R4C instrument has the potential to address these needs. The web-based application of the R4C instrument is easily administered, enables the use of components that reflect clinical reasoning in reality as well as providing objective and secure scoring, which is difficult to achieve when assessments are conducted in clinical practice [18],[19]. The R4C instrument may serve as a useful tool to determine physiotherapists' clinical reasoning at a group level and to evaluate educational interventions. The R4C instrument could be used for learning purposes, such as inclusion in formative assessments. Assessments have an impact on students' learning, as they often adapt what is learned according to what they believe will be assessed [71]. Hence, the R4C instrument may influence students' learning of clinical reasoning towards a biopsychosocial perspective and the integration of behaviour change strategies. However, the use of the R4C instrument in high-stake examinations requires further testing.

Use of the R4C instrument has implications for educators, researchers, and professional organisations because the instrument targets prioritised competence areas for the physiotherapy profession. By assessing the ability to make behavioural considerations throughout the reasoning process, the R4C instrument may contribute to a deeper understanding of clinical practitioners' and students' readiness to support clients in health-related behaviour changes. In addition, variations in the scores of the R4C instrument could prompt a closer look at the scores far from, or close to, the criterion scores to better understand more or less demanding elements in the reasoning process. Such investigations would provide valuable feedback to professional representatives and educators and could help inform learning activities and curriculum development. Psychometrically sound and effective clinical reasoning assessment tools are in demand across health professional education programmes. Therefore, the R4C instrument may be of interest to researchers and educators across health professions. The R4C instrument is based on Script concordance testing [21] and the Key features approach [32], which are established clinical reasoning instruments in the context of nursing and medicine. With this common foundation, the R4C instrument may provide instrument developers with new ideas and insights about evolving content and item construction. Furthermore, to achieve the global targets for the prevention and control of non-communicable diseases [1] health-related and lifestyle behaviour change needs to be initiated and supported across health professionals which, in turn may direct their clinical reasoning. To serve specific professional demands, adaptations of cases and item content in the R4C instrument may be required.

## 5. Conclusion

The web-based application of the previously established R4C instrument, assessing physiotherapists' clinical reasoning in integration with clients' behaviour change, shows satisfactory reliability and construct validity, and could be useful in evaluation in physiotherapy education and research. The developed criterion scores for items of the R4C instrument may contribute to the accurate interpretation of future test scores. Even though the results of the instrument are promising, there is a need for its further assessment. Future research is needed involving further evaluation of validity evidence, such as validation with contrasted groups, and testing of the R4C instrument with larger sample sizes and across various contexts.
