# Supplementary material for: Criterion scores, construct validity and reliability of a web-based instrument to assess physiotherapists' clinical reasoning focused on behaviour change: ‘Reasoning 4 Change’
Source: AIMS Public Health. 2018 Jul 6;5(3):235–59. doi: 10.3934/publichealth.2018.3.235 (PMC6141557; doi:10.3934/publichealth.2018.3.235)
Supplement: Supplementary file 1 [file publichealth-05-03-235-s001.pdf]

Public Health

AIMS Public Health, 5(3): 235–259.
DOI: 10.3934/publichealth.2018.3.235
Received: 28 April 2018
Accepted: 29 June 2018
Published: 06 July 2018

http://www.aimspress.com/journal/aimsph

Research article

Criterion scores, construct validity and reliability of a web-based instrument to assess physiotherapists' clinical reasoning focused on behaviour change: 'Reasoning 4 Change'

Maria Elvén[1,*], Jacek Hochwälder[2], Elizabeth Dean[1,3], Olle Hällman[4] and Anne Söderlund[1]

[1] Division of Physiotherapy, School of Health, Care and Social Welfare, Mälardalen University, Västerås, Sweden
[2] Division of Psychology, School of Health, Care and Social Welfare, Mälardalen University, Eskilstuna, Sweden
[3] Department of Physical Therapy, Faculty of Medicine, University of British Columbia, Vancouver, BC, Canada
[4] Department of Information Technology, Uppsala University, Uppsala, Sweden

* **Correspondence:** Email: maria.elven@mdh.se; Tel: +4621103109.

Supplement A. Examples of domain items in the Reasoning 4 Change instrument.

Domain 1 Physiotherapist

D1.1 Knowledge

Below are descriptions of the knowledge that physiotherapists can use in clinical reasoning. To what extent do you agree with the following statements? Select the option (1-6) that best matches your opinion.

	**Do not agree at all**					**Completely agree**
#1 I have very good knowledge of theories and models about how behaviours are learned (e.g. operant and respondent learning)	☐ 1	☐ 2	☐ 3	☐ 4	☐ 5	☐ 6
#8 I know which methods are effective for supporting clients' behavioural change based on my <u>own practical</u> experience	☐ 1	☐ 2	☐ 3	☐ 4	☐ 5	☐ 6

D1.2 Cognition

Below are descriptions of the cognitive capabilities and skills that physiotherapists can use in clinical reasoning. To what extent do you agree with the following statements? Select the option (1-6) that best matches your opinion.

	**Do not agree at all**					**Completely agree**
#4 I have good skills in analysing how the client's <u>physical and social environment</u> affect the performance of the target behaviour	☐ 1	☐ 2	☐ 3	☐ 4	☐ 5	☐ 6
#7 I have a good capability to formulate hypotheses (make assumptions) explaining how physical, psychological and environmental factors and consequences of the behaviour <u>are interrelated, cause and control</u> the client's difficulties in performing the target behaviour	☐ 1	☐ 2	☐ 3	☐ 4	☐ 5	☐ 6

D1.3 Metacognition

Below are descriptions of the metacognitive capabilities and skills that physiotherapists can use in clinical reasoning. To what extent do you agree with the following statements? Select the option (1-6) that best matches your opinion.

	**Do not agree at all**					**Completely agree**
#4 I re-evaluate my hypotheses (assumptions) about what influences the client's target behaviour when I am uncertain about their accuracy	☐ 1	☐ 2	☐ 3	☐ 4	☐ 5	☐ 6
#6 When implementing treatment, I often ask myself if I have considered all possible treatment strategies to help the client achieve their target behaviour	☐ 1	☐ 2	☐ 3	☐ 4	☐ 5	☐ 6

D1.4 Psychological factors

Below are descriptions of methods that physiotherapists in their clinical reasoning can choose to use in their practical work.

How <u>**important**</u> do <u>**you**</u> think it is for you <u>**to use**</u> these methods in your practical work?
Base your responses on how important you feel the method is for you to use, not on your ability. Select the option (0-10) that best matches your opinion.

	**Not very important**										**Extremely important**
#5 Guiding the client to independently monitor their target behaviour in its natural context, e.g. through a diary	☐ 0	☐ 1	☐ 2	☐ 3	☐ 4	☐ 5	☐ 6	☐ 7	☐ 8	☐ 9	☐ 10
#8 Together with the client formulate SMART goals for treatment. SMART = specific, measurable, activity-related, realistic and time-specific	☐ 0	☐ 1	☐ 2	☐ 3	☐ 4	☐ 5	☐ 6	☐ 7	☐ 8	☐ 9	☐ 10

How <u>**certain are you that you can use**</u> the following methods in your practical work?
Select the option (0-10) that best matches your opinion.

	**Not Certain at all**										**Highly certain**
#15 Guiding the client to independently monitor their target behaviour in its natural context, e.g. through a diary	☐ 0	☐ 1	☐ 2	☐ 3	☐ 4	☐ 5	☐ 6	☐ 7	☐ 8	☐ 9	☐ 10
#18 Together with the client formulate SMART goals for treatment. SMART = specific, measurable, activity-related, realistic and time-specific	☐ 0	☐ 1	☐ 2	☐ 3	☐ 4	☐ 5	☐ 6	☐ 7	☐ 8	☐ 9	☐ 10

D1.5 Contextual factors

Below are descriptions of workplace-related factors that may be of significance for physiotherapists' clinical reasoning. To what extent do you agree with the following statements? Select the option (1-6) that best matches your opinion.

If you are a physiotherapy student, base your responses on your placement (clinical training placement) over the past six months. If you are a physiotherapist, base your responses on your current workplace.

	Do not agree at all					Completely agree
#2 There are factors at my clinical training placement/workplace, that make it difficult for me to focus on clients' target behaviour and behavioural change in my clinical reasoning.	☐ 1	☐ 2	☐ 3	☐ 4	☐ 5	☐ 6
#4 I feel that my supervisors/colleagues focus on clients' target behaviour and behavioural change in their clinical reasoning.	☐ 1	☐ 2	☐ 3	☐ 4	☐ 5	☐ 6

Domain 2 Input from client

Case description: Joseph, age 48

Joseph is a 48-year-old man who has had lumbar pain for the past six months. The pain began slowly at first but has since gradually increased. Joseph has pain at rest in the lumbar region, movement-related pain on the left side of his back and radiating pain towards the back of his left thigh. He often wakes up during the night because of the pain but changing his sleeping position alleviates the pain and he can fall asleep again. Five years ago, Joseph suffered a right L4-L5 disc herniation and he had major difficulty performing even simple activities such as light household activities and gardening. He was on sick leave for eight months during this time. Surgery was eventually performed on the herniated disc and the worst pain disappeared. Now Joseph wonders if he has a new herniated disc in his back. Joseph works full-time in a customer reception, which involves a lot of sitting, which is difficult. He used to take daily walks with his wife, but stopped taking walks completely a couple of months ago due to the increasing discomfort in his back.

Item 1: What three factors in the interview/case history do you think are the most important to ask more about in your first consultation in order to understand Joseph's complaints and situation?
Select three of the underlined factors in the case description.

Factor 1:________________________________ Factor 2:____________________________________ Factor 3:________________________________

Item 2: Based on what you now know about Joseph, you need to collect more information in your interview/case history to understand his complaints and situation. Several areas can be important to get more information about, but which area would you prioritise as most important?
Select the option (only one) that you think is most important.

☐*Physical and biomedical factors*	Joseph's description of his physical resources and limitations and/or biomedical factors that are of significance for his complaints and situation.<br>For example, strength, balance, mobility, pain, breathing and condition, as well as pathological or pathophysiological changes, e.g. pain, sensitivity, ligament and muscle injury
☐*Psychological factors*	Joseph's description of his psychological resources and limitations that are of significance to his complaints and situation.<br>For example, thoughts, feelings, beliefs, expectations, fear and coping.
☐*Contextual factors*	Physical and social environmental factors and/or personal factors that may affect Joseph's complaints and situation.<br>For example, assistive devices, exercise equipment, friends and relatives, and the client's sociocultural backgroun
☐*Behavioural skills*	Joseph's description of his ability to perform a target behaviour in an everyday activity.<br>A defined target behaviour in an everyday activity that the client experiences as difficult and which hinders the performance of the activity. Target behaviour is something that the client feels is important to be able to manage. For example, putting on their socks, working at their computer or running on uneven ground.
☐*Lifestyle-related risk factors*	Joseph's health-related living habits that are of significance to his complaints and situation.<br>For example, physical activity, smoking, alcohol consumption habits and eating habits.

Domain 3 Functional behavioural analysis

Case description: Anna-Lena, age 75

Anna-Lena is 75 years old and lives alone in a two-storey house. She reads a lot during the days and she is involved with the Red Cross. She is healthy but feels that over the past few years, she has less energy and taking care of the house is beginning to get difficult. For the past six months, she has experienced weight-bearing pain in her left knee that worsens the

more she bends her knee. There is no known trauma, however the discomfort has progressed gradually. The discomfort in her knee is now so great that it influences her daily life. Her walking ability has been greatly affected and she can now only walk 1.5 km. She has great difficulty walking on steep hills and walking up and down stairs. It is especially difficult to go down the steep spiral staircase between the floors of her home. She has fallen on the stairs several times in the past month when she experienced a sensation that her knee locked and she lost her balance.

Her left knee is slightly swollen on the inside and she has reduced balance when standing on her left leg. The knee snaps and locks and Anna-Lena doesn't trust the knee. Anna-Lena is used to managing on her own and is irritated that the knee is limiting her.

When Anna-Lena takes the stairs, she tenses up so that she will be prepared if her knee locks up. In the morning, when it's a bit dark and she's just woken up and has to go downstairs, she feels wobbly and it's particularly difficult to walk. She is worried that she will injure herself and not be able to get up on her own if she falls again. She increasingly avoids taking the stairs so that she won't expose herself to the risk of falling.

Anna-Lena's *target behaviour*: *Walking safely up and down the spiral staircase at home.*

Item 1: Based on the information you now have about Anna-Lena, what three hypotheses/assumptions do you think explain <u>the most important causes</u> for her difficulty performing the target behaviour? Select three hypotheses:

☐ 1. The difficulty walking on the spiral staircase is caused by probable knee osteoarthritis.

☐ 2. The difficulty walking on the spiral staircase is caused by a fear of falling, resulting in Anna-Lena largely avoiding taking the stairs at home.

☐ 3. The difficulty walking on the spiral staircase is caused by her knee locking, which inhibits muscle control and leads to a feeling of instability.

☐ 4. The difficulty walking on the spiral staircase is caused by the staircase being so steep that her knee needs to be significantly flexed, which causes more pain.

☐ 5. Anna-Lena's ability to manage to walk on the spiral staircase is encouraged by her strong will to fend for herself.

☐ 6. The difficulty walking on the spiral staircase is caused by a decreased ability to walk when it is dark because her balance is impaired and she feels unsteady and uncertain.

Item 2: All six hypotheses/assumptions are listed separately below. Each hypothesis explains one cause for Anna-Lena's difficulty performing her target behaviour. In your consultations with Anna-Lena, you have subsequently acquired new information that may affect the hypothesis. You will now determine if the new information strengthens or weakens the stated hypothesis/assumption.

Select the number that best matches your opinion.

Hypothesis/assumption explaining the cause for Anna-Lena's difficulty performing her target behaviour.	***…… and if you get new information that ……***	***… do you think that this strengthens or weakens the first hypothesis/assumption?***	
1. The difficulty walking on the spiral staircase is caused by probable knee osteoarthritis.	Physical examination shows full active and passive mobility in the left knee and bilateral muscle strength in the quadriceps.	**- 2**	The hypothesis greatly weakens
		**- 1**	The hypothesis somewhat weakens
		**0**	The hypothesis neither weakens or strengthens
		**+1**	The hypothesis somewhat strengthens
		**+2**	The hypothesis greatly strengthens

2. The difficulty walking on the spiral staircase is caused by a fear of falling, resulting in Anna-Lena largely avoiding taking the stairs at home.	Anna-Lena rates 2 on how certain she is about walking down the stairs at home on a scale of 0 = not at all certain to 10 = very certain.	- 2<br>- 1<br>0<br>+1<br>+2	The hypothesis greatly weakens<br>The hypothesis somewhat weakens<br>The hypothesis neither weakens or strengthens<br>The hypothesis somewhat strengthens<br>The hypothesis greatly strengthens

3. The difficulty walking on the spiral staircase is caused by her knee locking, which inhibits muscle control and leads to a feeling of instability.	Anna-Lena feels that the best thing she can do to prevent the pain and ache in her knee from increasing is to be careful with all unnecessary movements of the knee.	- 2<br>- 1<br>0<br>+1<br>+2	The hypothesis greatly weakens<br>The hypothesis somewhat weakens<br>The hypothesis neither weakens or strengthens<br>The hypothesis somewhat strengthens<br>The hypothesis greatly strengthens

4. The difficulty walking on the spiral staircase is caused by the staircase being so steep that her knee needs to be significantly flexed, which causes more pain.	Anna-Lena's own self-monitoring through a diary reveals that she thinks it is easier to walk down the stairs with her left leg first.	- 2<br>- 1<br>0<br>+1<br>+2	The hypothesis greatly weakens<br>The hypothesis somewhat weakens<br>The hypothesis neither weakens or strengthens<br>The hypothesis somewhat strengthens<br>The hypothesis greatly strengthens

5. Anna-Lena's ability to manage to walk on the spiral staircase is encouraged by her strong will to fend for herself.	Anna-Lena doesn't think that physiotherapy will reduce her pain in any significant way.	- 2<br>- 1<br>0<br>+1<br>+2	The hypothesis greatly weakens<br>The hypothesis somewhat weakens<br>The hypothesis neither weakens or strengthens<br>The hypothesis somewhat strengthens<br>The hypothesis greatly strengthens

6. The difficulty walking on the spiral staircase is caused by a decreased ability to walk when it is dark because her balance is impaired and she feels unsteady and uncertain	The steps of the spiral staircase are made of wood and when Anna-Lena walks down them in the morning, she is usually wearing knitted socks.	- 2<br>- 1<br>0<br>+1<br>+2	The hypothesis greatly weakens<br>The hypothesis somewhat weakens<br>The hypothesis neither weakens or strengthens<br>The hypothesis somewhat strengthens<br>The hypothesis greatly strengthens

Domain 4 Strategies for behaviour change

The case description begins with a hypothesis/assumption. The hypothesis/assumption describes conclusions on how different factors affect the client's ability to perform the target behaviour in an activity that she wants to be able to better manage.

Case description: Samira, age 37
Samira's target behaviour: Performing regular physical activity by walking 30 minutes a day.

Hypothesis/assumption
Samira has been physically inactive for the past 10 years. The difficulties performing regular physical activity by walking are related to tension headaches, localised to the forehead and temples, which means that she doesn't have the energy to go out. The headaches are associated with increased tone, tightness and tenderness in the suboccipital and trapezius muscles. Her headaches are aggravated by stress at work. When she gets a headache, she rests or takes a painkiller, but this doesn't always help. She therefore lacks good strategies to reduce the pain. The physical inactivity is also due to several unsuccessful attempts to walk regularly and for a long period of time. It has been difficult for her to find time in her day and she hasn't found it enjoyable or worth the effort. This gives her little confidence in her ability to manage it this time. Three years ago, Samira was diagnosed with diabetes type 2. Her lack of knowledge about the benefits of physical activity for diabetes also contributes to her low level of activity.

Item 1: Do you believe that Samira's difficulty performing her target behaviour right now is mainly due to physical/biomedical, psychological or environmental factors (physical and/or social)?
Indicate the importance of these factors by assigning a percentage to each of these factors (the percentages should add up to 100%).
E.g. Physical/biomedical factors 30%; psychological factors 10% and environmental factors 60%.

	**Per cent %**
**Physical/biomedical factors**	
**Psychological factors**	
**Environmental factors (physical and/or social)**	

Item 2: To help Samira achieve her target behaviour, you need to use different treatments/interventions. What four treatments/interventions do you think are most important at this stage and those you want to prioritise to begin with?

Formulate your answers as briefly as possible. No more than 10 words per answer.

1.

2.

3.

4.

More information about Samira

You have now consulted with Samira a few times and she has taken walks directly after work four times in the last week. However, she isn't quite satisfied. She wants to begin exercising, but she also wants to pick up her children from their after-school programme as soon as possible after work.

Samira's target behaviour: Performing regular physical activity by walking 30 minutes a day.

Item 3: Below are treatments/interventions to support Samira achieve her target behaviour. In your consultation with Samira, you acquire new information that may affect your choice of treatment/intervention. You should now determine how this new information influence your choice of treatment/intervention.

Select the number that best matches your opinion.

You think that a relevant treatment/intervention would be to ………..	***…… and then you receive new information that ……***	***….how do you then assess the proposed treatment/intervention?***

Increase positive outcome expectations through graded activity	At follow-up one week after this treatment/intervention, Samira says that she has taken two short evening walks this week after the children have fallen asleep. It feels good that the exercise is not affecting her time with her children.		**The treatment/intervention was:**
		**- 2**	Very irrelevant
		**- 1**	Somewhat irrelevant
		**0**	Neither relevant or irrelevant
		**+1**	Somewhat relevant
		**+2**	Very relevant

Manual therapy/treatment for improved suboccipital and trapezius muscle function	Samira experiences a fear of performing moderately strenuous physical activity, e.g. brisk walking and cleaning. She rates this at 7 on a scale of 0-10 (0=not at all afraid and 10= extremely afraid)		**The treatment/intervention is:**
		**- 2**	Very irrelevant
		**- 1**	Somewhat irrelevant
		**0**	Neither relevant or irrelevant
		**+1**	Somewhat relevant
		**+2**	Very relevant

The R4C instrument includes 79 items, distributed as follows: Domain 1.1 Knowledge comprises 8 items, Domain 1.2 Cognition comprises 8 items, Domain 1.3 Metacognition comprises 8 items, Domain 1.4 Psychological factors comprises 20 items, Domain 1.5 Contextual factors comprises 5 items, Domain 2 Input from client comprises two cases with six items per case, Domain 3 Functional behavioural analysis comprises four cases with two items per case, and Domain 4 Strategies for behaviour change comprises two cases with five items per case.

More information regarding the domains of the R4C instrument and characteristics of cases, items and response scales are provided in: Elvén M, Hochwalder J, Dean E, Söderlund, A (2018) Development and initial evaluation of an instrument to assess physiotherapists' clinical reasoning focused on clients' behavior change. Physiother Theory Pract 34: 367-383.

Supplement B. Design and system requirements and related features in layout and function of the web-based application of the Reasoning 4 Change instrument.

Design and system requirements[a]	Features in layout and function
Easy access to the instrument	Access is provided via a web-link. At any point, the user is able to pause progress and continue at a later, more convenient time and/or with a more convenient device (e.g. the user starts off with a PC and later finishes on a tablet).
Secure personal log-in	The user is provided with a personal ID code and selects a personal user name and password. All passwords are stored in an encrypted format.
Provide the user with clear instructions and definitions of key concepts and arrange for reading request	A clickable button is used to confirm that the instructions and definitions have been read. A word list, including instructions and definitions is available anywhere in the instrument.
Disable the ability to change a decision already made in the reasoning process	When the submit button is pressed for a set of items, the provided responses are locked.
Limit information displayed on the screen to support simplicity	In the long list of strategies for behaviour change in Domain 4, descriptions and examples are concealed and are only displayed when the drop-down list of options is activated. Previous descriptions of a case are concealed but are always available in pop-up menus.
Controlling the response process	Controlling for correct answering procedure: Slider controls are used to provide responses in percentages, thereby controlling a maximum of 100%. All items and required response options must be answered to submit and proceed. The selection of more response options than requested is not possible. Write-in answers are regulated by a maximum word count.
Facilitate administration	An administration tool is included in the web application which enables administration and charting of items, responses, user profiles etc.
Facilitate the scoring process	Scores of individual items and total scores for subscales and domains are automatically generated and exported as files that meet the requirements for statistical analyses.
Facilitate instant reinforcement	The user interface provides positive feedback when the user completes a domain. As the user completes a set of items within a domain, progress is shown. Visible immediate results enhance the user experience (Tidwell, 2011). The interface provides an easy and fast way to start or continue the instrument when logging in to reduce time spent on navigation. Additional measures to facilitate instant reinforcement include an authentication system that allows the user to remain logged in while the browser tab or the browser itself is closed, thus, making it faster to resume completing the instrument.

Facilitate safe exploration	The user interface is designed so that users can safely explore its various parts without consequences of data loss in the form of lost user responses. A user may rapidly scan the interface and choose the first option that she decides is in line with her goal, even if it is the wrong action (Tidwell, 2011). Thus, the interface is designed to handle user mistakes in an intuitive manner.
Reduce cognitive cost	Visual simplicity and plainly worded labels are provided throughout the interface. Menu items in the top bar contain both icons and text to increase recognition.
Facilitate interface consistency	Information and controls are placed in consistent locations. As the user navigates through the domains with different form elements, the overall layout, interface design and actionable elements remain similar. The domain elements are placed in consistent locations with identical aesthetic styles. These design choices prevent user errors caused by biased perception (e.g. habituation) (Johnson, 2014).
Provide strong visual structure and distinct information presentation	Input forms and information presented to the user contains clear visual structure. Text is appropriately formatted, visual noise is reduced and form elements enhance visual structure (e.g. striped tables, percentage sliders). Colours throughout the interface are selected to facilitate clear text, forms and navigation. Orange is used for information, blue for clickable buttons, and warm yellow, blue green, purple and green to distinguish the domains. If information and forms are correctly visually structured, scanning and comprehension is made easier and quicker (Johnson, 2014).

[a]What the application should provide for or do.

Johnson J (2014) *Designing with the mind in mind*, 2 Eds., Amsterdam: Morgan Kaufmann, Elsevier Inc.

Tidwell J (2011) *Designing interfaces: Patterns for effective interaction design*, 2 Eds., Sebastopol: O'Reilly Media, Inc.
